# Favorable Outcome with Close Margins in Patients Undergoing Nipple/Skin Sparing Mastectomy with Immediate Breast Reconstruction: 5-year Follow-up

**DOI:** 10.4274/balkanmedj.2017.0029

**Published:** 2018-01-20

**Authors:** Enver Özkurt, Mustafa Tükenmez, Erdem Güven, Burcu Çelet Özden, Gizem Öner, Mahmut Müslümanoğlu, Abdullah İğci, Vahit Özmen, Seden Küçücük, Neslihan Cabioğlu

**Affiliations:** 1 Department of General Surgery, Breast Unit, İstanbul University İstanbul School of Medicine, İstanbul, Turkey; 2 Department of Plastic Surgery, İstanbul University İstanbul School of Medicine, İstanbul, Turkey; 3 Department of Radiation Oncology, İstanbul University İstanbul Institute of Oncology, İstanbul, Turkey

**Keywords:** Breast implants, mastectomy, subcutaneous, breast reconstruction, surgical margin, local recurrence

## Abstract

**Background:**

Implant-based breast reconstruction after mastectomy has recently been reported to be the preferred type of surgery among breast-specific surgeons and plastic surgeons.

**Aims:**

To explore the significant clinicopathological factors associated with long-term outcome related to local recurrences of the nipple among patients who underwent immediate breast reconstruction with tissue expander or implant after mastectomy.

**Study Design:**

Retrospective cohort.

**Methods:**

From January 2007 to January 2013, 51 breast cancer patients who underwent immediate breast reconstruction with tissue expander or implant were retrospectively analysed. Patients’ demographic data, clinicopathological characteristics, and clinical outcome by disease-free survival and disease-specific survival analyses were determined.

**Results:**

The median follow-up was 64 (31-114) months. Of the 57 mastectomies, 41 were skin sparing mastectomy (72%) and 16 were nipple-areola sparing mastectomy (28%). Immediate breast reconstruction surgery included tissue expander (n=46, 81%) or implant (n=11, 19%) placement. The molecular subgroups of 47 invasive cancers were as follows: luminal A (n=23, 49%), luminal B (n=16, 34%), non-luminal HER2 (n=5, 10.6), triple negative breast cancer (n=3, 6.4%). The 5-years disease-specific survival, disease-free survival, and locoregional recurrence-free survival rates were 96.8%, 90%, and 97.6% respectively. Patients with luminal A cancer were found to have an improved 5-year disease-free survival time than other (luminal A; 100% vs. non-luminal A; 78%; p=0.028). Of the 14 nipple-areola sparing mastectomy, 13 had a close median tumour distance to nipple-areola complex (<20 mm) with a 5-year locoregional recurrence free survival of 100%.

**Conclusion:**

Immediate breast reconstruction with implant or tissue expander can be safely applied in patients undergoing skin sparing mastectomy or nipple-areola sparing mastectomy. Patients with luminal-A type show the most favourable outcome. During the 5-year follow-up period, patients even with close margins (<20 mm) to nipple-areola complex with nipple-areola sparing mastectomy have excellent locoregional and overall survival when treated by contemporary multidisciplinary oncological management.

Mastectomy is performed in approximately 40-50% of women with breast cancer (1). Implant-based breast reconstruction after mastectomy has recently been reported to be the preferred type of surgery among plastic surgeons comprising approximately 79% of all breast reconstruction procedures in the USA (2).

Skin-sparing mastectomy has resulted in higher levels of patient satisfaction and quality of life (3). Nipple-areola-sparing mastectomy (NSM), in which the nipple-areola complex (NAC) is preserved, coupled with reconstruction, can result in remarkable aesthetic results, with the improved techniques related to breast implants, acellular tissue matrices, and fat grafting (4). The most important advantages of skin sparing mastectomy (SSM) or nipple-areola sparing mastectomy (NSM) are the preservation of the breast contour, preservation of the submammary fold, and avoidance of skin differences.

Many studies comparing local and distant recurrence in patients undergoing SSM with those undergoing conventional mastectomy have reported no significant differences, thus supporting the oncological safety of this approach (5,6). Although there are some controversies about the risk of local relapse, various clinical studies have shown that the NSM also is a safe procedure for selected cases (7,8,9,10). These results have further been maintained in long-term outcome studies, where NSM shows oncologic outcomes similar to SSM (10,11). The locoregional recurrence (LRR) rate after NSM ranges from 0 to 11.7% (8,11,12,13,14,15). The recurrence in the NAC is a rare event with incidence of 0-5%. Besides, considering that a LRR is more likely to occur within the first two years after primary surgery, the length of follow-up is also important (16).

Our aim in this study has been to determine the significant clinicopathological factors associated with long-term outcome among our institutional patient cohort who underwent immediate breast reconstruction (IBR) with tissue expander (TE) or implant after mastectomy treated with contemporary oncological management.

## MATERIALS AND METHODS

### Patients

Between January 2007 and January 2013, 51 patients with a diagnosis of breast cancer underwent IBR with implant or TE. Fifty-seven mastectomies and IBR with implant or TE were performed in 51 patients. Patients’ demographic data were collected from breast charts and were reviewed for age, history of the disease, family history, medical history, smoking status, physical examination findings (clinical stage), cancer characteristics (cancer type, hormone receptor status, HER2/neu status, etc.), pathological stage, reconstruction type (TE or implant), preoperative systemic chemotherapy and adjuvant treatment (chemotherapy, radiotherapy, hormonal therapy), complications and any systemic or local recurrence (LR). Major complications were defined as any complication that necessitated a repeat surgical procedure such as implant or TE removal. This study was approved by Scientific and Ethical Committee of İstanbul University İstanbul School of Medicine (2017/656).

### Surgical technique

Sentinel lymph node biopsy was performed either with blue dye (patent blue) and/or radiocolloid injection by using gamma probe at the discretion of the surgeon. After the induction of general anaesthesia, blue dye was applied to the deep subareolar tissue followed by breast massage for 5 minutes. Axillary lymph node dissection was performed in patients with a positive finding in intraoperative pathological assessment of the sentinel lymph nodes. Surgery was decided as SSM for patients whose NAC is identified as involved by tumour either with radiologic assessment or intraoperative pathological assessment. Otherwise NSM is selected.

The mammary incision was carried out by elevating the skin flaps in the same planes. Subdermal tissue with its vascular plexus was separated from breast tissue. Depending on the patient, a 2-5 mm thickness of posterior areolar tissue was left, aiming to assure the viability of the nipple areola complex. At this time, another intraoperative pathological assessment was performed from the posterior part of the nipple for cases with NSM. If the result was positive, the nipple-areola complex was removed. After circumferential elevation of the skin flaps, shaving off the pectoral fascia was performed, and the specimen was delivered out of the skin pocket. Following SSM or NSM, the pectoralis major muscle was elevated starting from its lateral border and both sternal and inferior attachments below the inframammary fold were released with meticulous dissection that allowed the surgeon to stay strictly within the submuscular plane. In addition, the serratus fascia was also elevated laterally to accommodate the lateral border of the implant or TE. After copious irrigation of the implant pocket and insertion of two (submuscular or subdermal) drains, the implant or TE was placed submuscularly. We prefer to use TE for the first-stage reconstruction if the patient was considered to receive postoperative radiotherapy. Otherwise, we prefer to use directly implant. The lateral border of the pectoralis and serratus muscle was sutured to allow for the complete coverage of the device. If the TE was used, it was inflated perioperatively to approximately half to 2/3 of its final volume, depending on the perfusion of the mastectomy flaps.

Antibiotic prophylaxis was started with 4 g ampicillin/sulbactam daily and was continued until the drains were removed if the drainage was less than 30 cc daily. TE inflation was started on the third postoperative week and an average of 3 sessions was performed to reach the desired TE size before the start of radiotherapy. Radiotherapy was planned with TE in the fully inflated state. Exchange to implant with additional fat grafting to the breast pocket was performed between 2 and 8 months following radiotherapy.

### Statistical analysis

All statistical analyses were performed using SPSS 17.0 for Windows software (SPSS Inc., Chicago, IL, USA). The Kaplan-Meier test was used to calculate the overall survival (OS), Disease-specific survival (DSS), disease-free survival (DFS), and LRR free survival rates. All p values were two-sided, and a p-value less than 0.05 considered as a statistically significant difference. The statistical and/or clinically significant variables were further evaluated by Cox regression analysis.

## RESULTS

Patient demographics and treatment modalities are shown in Table 1. The median age was 42 (range, 20-74). Eighteen patients had a family history of breast cancer (35%). Among the 11 patients who were tested for a *BRCA* mutation due to a strong family history of breast cancer (blood relative with known *BRCA* mutation, blood relative with two or more primary breast cancer, two or more relatives with breast cancer on the same side of the family, blood relative with ovarian cancer, blood relative with male breast cancer, patients under 40-year old that demonstrate triple negative molecular subtype), only 1 patient was found to have a *BRCA1* mutation. Thirty-nine patients were premenopausal (76.5%), 9 were postmenopausal (17.6%) and 3 were perimenopausal (5.9%).

Of the 57 mastectomy procedures, 6 were bilateral. Indications for bilateral mastectomy were bilateral breast cancer (n=4), the presence of a *BRCA1* mutation (n=1), and the presence of high risk lesions such as atypical ductal hyperplasia in the contralateral breast (n=1). Surgical type of mastectomy was SSM in 41 cases (72%) and NSM in 16 cases (28%), including 2 cases with video endoscopic assistance. Two of them were prophylactic NSM. A tissue expander was used in 46 (81%) mastectomies, whereas an implant was placed in 11 (19%) mastectomies.

Tumour characteristics are summarised in Table 1. Most of the cases were T1/T2 (72.5%). Twenty-five (49%) patients were pathologically lymph node positive. The majority of the tumours were grade II/III (70.6%). Thirty-one (61%) patients were stage 2. Of 47 patients with invasive breast cancer, 23 were luminal A (48.9%), 16 were luminal B (34%), 5 were non-luminal HER2 (10.6%) and 3 were triple negative breast cancer (6.4%). No statistical difference was detected between patient distribution of luminal A and luminal B subtype according to the pathological stage with primary surgery or clinical stage for the patients with preoperative systemic chemotherapy [Luminal A; stage 1 (n=4), stage 2 (n=14), stage 3 (n=3) vs. luminal B; stage 1 (n=3), stage 2 (n=10), stage 3 (n=3), p=0.977].

Six patients received preoperative systemic chemotherapy (11.8%), whereas 32 patients had adjuvant chemotherapy (62.7%). The remaining 13 (25.5%) did not receive any chemotherapy. All of the patients were discussed by the tumour board and 22 patients (48%) chose to receive post-mastectomy irradiation (3 patients with implant replacement) and 37 patients (72.5%) selected adjuvant hormonotherapy. As a policy, the majority of the patients who were treated with chest wall irradiation (n=19; 86%) received TE placement after preoperative systemic chemotherapy or before adjuvant chemotherapy to be replaced with permanent implants followed by radiotherapy (Table 1).

Major and minor complications following mastectomy with IBR occurred in 11 of 57 mastectomies (19.3%). Eight patients (14%) had unilateral implant or TE loss (n=6 in SSM, n=2 in NSM group). There were only 3 (5%) implant or TE losses due to radiotherapy. Partial NAC necrosis was seen in 2 of 16 NSM (12%); only one needed a partial debridement. None of our patients had a complete nipple areola complex necrosis. The other complications were wound infection in 4 patients (7%), partial dehiscence in 1 patient (1.8%), skin flap necrosis in 1 patient (1.8%), puncture of the expander in 1 patient (1.8%), and implant or TE exposure in 8 patients (14%).

**Figure 1:**
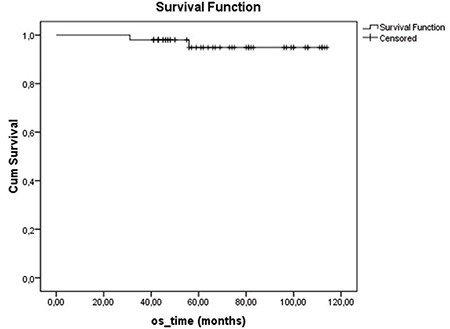
Overall survival of 51 patients. *OS: overall survival*

**Figure 2:**
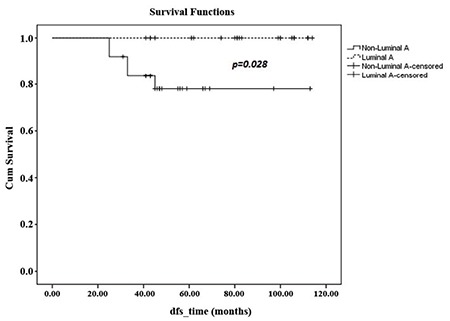
Disease-free survival of Luminal A and non-Luminal A patients. *DFS: disease-free survival*

**Figure 3:**
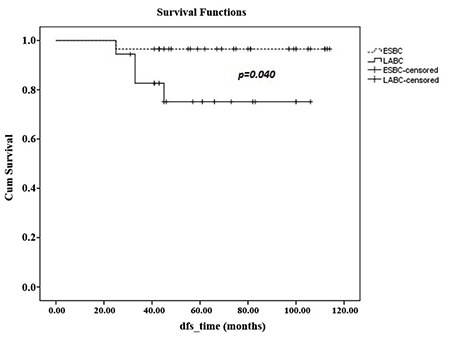
Five year disease-free survival of early stage breast cancer and locally-advanced breast cancer patients. *DFS: disease-free survival; ESBC: early stage breast cancer; LABC: locally-advanced breast cancer*

At a median follow-up period of 64 months (range, 31-114), 1 had LR along with systemic metastases in the 45th month of follow-up, with an LR rate of 1.8% in our series. The patient with LR underwent SSM, and had a grade III luminal B tumour type. The patient died in the 56th month due to multiple organ metastases and multiple organ dysfunctions. Totally, systemic disease was determined in 5 patients during their follow-up (9.8%). The 5-year OS of 51 patients was 95% (Figure 1). Five year DFS, DSS, and LRR free survival of patients were 90%, 96.8%, and 97.6%, respectively. Furthermore, factors associated with survival including age (<50, ≥50 and <40, ≥40), median tumour size (25 mm), lymph node status, pathological stage, early stage breast cancer (ESBC) and locally advanced breast cancer (LABC), tumour grade, lymphovascular invasion, oestrogen receptor and progesterone receptor status, HER2/neu status, and molecular subtype of the patients were analysed (Table 2). There was no significant difference in DSS analysis; however, the results of the DFS subgroup analysis revealed that patients with a tumour diameter smaller than 20 mm (p=0.045), grade I-II (p=0.026), luminal A molecular subtype (p=0.028, Figure 2), and ESBC (p=0.040, Figure 3) were more likely to have a better outcome (Table 2). Independent factors affecting DFS was also analysed in multivariate analysis. Multivariate cox regression analysis revealed that only patients with luminal A subtypes were more likely to have to have a better DFS rate (HR=1.4; 95% CI:1.4-8.2; p=0.007) (Table 3).

Of the 14 NSM with invasive cancer, intraoperative pathological assessment revealed no tumour involvement of the posterior part of the nipple, and the median tumour distance to NAC was 4.5 mm (1-50) at the final definitive pathological assessment. In our series, only one case had a tumour distance to NAC was ≥20 mm. One patient received preoperative systemic chemotherapy, 10 received adjuvant chemotherapy, and 3 did not receive any systemic treatment. Seven patients had adjuvant radiotherapy and 11 patients received adjuvant hormonotherapy. The median tumour size was 32.5 mm (8-70). Seven patients (50%) were LABC. Molecular subtypes of the patients were luminal A (n=4), luminal B (n=6), triple negative (n=1), and non-luminal HER2 (n=1). Five year DSS and LRR free survival rates were both 100%, whereas 5-year DFS and OS rates were 84.6% and 94.2%, respectively (Table 4).

## DISCUSSION

A tendency towards implant-based breast reconstruction continues to rise (17). In a study by Yi et al. (18), a total of 1810 breast cancer patients who underwent SSM (n=799, 44.1%) or conventional mastectomy (n=1011, 55.9%) were compared. The local, regional, systemic recurrence rates and DFS between two groups did not differ significantly, so they concluded that SSM is an acceptable option for patients who are candidates for IBR. Furthermore, in their meta-analysis, Lanitis et al. (19) found a 5.7% LR rate in SSM vs. 4% LR in non-SSM. This meta-analysis of 7 observational studies (825 SSM and 2518 non-SSM patients with no significant difference in stage or invasive cancers) also showed comparable survival rates between SSM and non-SSM (8.3% vs. 12.1%; OR= 0.63; 95% CI, 0.43-0.92). Similar to previous studies, our results also showed low LR rate as 1.8% as detected in one case with luminal B tumour after SSM along with systemic recurrence in our series. There was no LR in the NSM group.

Recent studies have shown that the NSM is a safe procedure for selected group of patients even in LABC patients (7,8,9,10,11,20,21,22). Chen et al. (23) evaluated 115 NSM and IBR with TE or silicone implant. The authors advocate that with two-stage reconstruction, it is possible to achieve the maximum control over the skin flap and by limiting the volume of the TE such that the skin envelope is not redundant; the risk of ischaemia is reduced. In addition, future aspects relating to NAC position, asymmetry and implant asymmetry can be managed at the time of the replacement of the TE with a silicone implant. In our series, if the patient was considered to receive postoperative radiotherapy, we preferred to use TE for the first-stage reconstruction (n=46, 81%). Otherwise, we preferred to use directly implant (n=11, 19%) for immediate breast reconstruction.

Peled et al. (22) published the largest series of LABC patients who treated with NSM and immediate reconstruction by implant or TE. During the mean follow-up period of 41 months, their LR, distant recurrence, and simultaneous local and distant recurrence rates were 5%, 15.1%, and 2.2% respectively, and the DFS was reported as 70%. They mentioned that patients with LABC are most at risk for distant recurrence rather than LR. Even after NSM, it is not associated with an increased risk for LR in this population. Similarly, we reported only one case with simultaneously local and distant recurrence with a rate of 1.8% in our series. Of the 47 patients with invasive cancer, 29 (61.7%) were ESBC and 18 (38.3%) were LABC. Similar to the findings published by Peled et al. (22), the 5-year DFS among patients with LABC was 75.1%.

Our systemic recurrence rate is 9.8% (5 of 51 patients) with a median follow-up of 5 years. Among 51 patients, our OS, DSS and DFS rate is 95%, 96.8% and 90% respectively. In the subgroup analysis of factors affecting DSS and DFS in our series; tumour size smaller than 20 mm (p=0.045), grade 1-2 (p=0.026), and luminal A molecular subtype (p=0.028) were found to be associated with more favourable outcome (Table 2). In a large series of 539 patients (565 breast cancers), Carlson et al. (24) reported LR of 5.5% (n=31) after a median follow-up of 61.6 months. LR rates were 0.6%, 3.0%, 10.4%, 11.1%, and 0% in stage 0, 1, 2, 3, and 4, respectively. In concordance with our findings, high tumour grade was significantly associated with recurrence.

In a multivariate analysis of 501 patient blocks and at a median follow-up of 10 years, Liu et al. (25) showed that luminal A subtype was significantly associated with low ipsilateral breast recurrence. In another study with a median follow-up of 12 years, 2985 patients’ tissue blocks were classified according to molecular subtype. Similarly, luminal A tumours were associated with a low risk of local or regional recurrence among the other subtypes in multivariate analysis (26). In a systematic review by receptor phenotype, 12.592 breast cancer patients who underwent either breast conserving surgery (n=7.174) or mastectomy (n=5.418) were identified from 15 studies by Lowery et al. (27). They assessed that luminal tumours exhibit the lowest rates of LRR. In concordance with these previous studies, we also found that Luminal-A type breast cancer undergoing SSM or NSM with IBR showed excellent survival outcome.

Essentially, some studies have considered NSM safe in patients with small, peripherally located tumours, without multicentricity, or for those who undergo risk reduction surgery (9). Although most of the clinical series emphasise that the principal selection criteria for NSM is the tumour to NAC distance, the ideal distance has not yet been clarified (28,29). Even though there is still no consensus about the selection criteria, most of the studies include tumour sizes up to 3 cm with a lack of clinical involvement of the NAC and a tumour to nipple distance greater than 2 cm (29). In a largest series of NSM that includes 981 mastectomies of 633 patients, with a median follow-up time of 29 (14-54) months, the authors highlighted that even in LABC, NSM can be performed (21). In their series, the overall 5-year cumulative incidences of LRR were 3% and distant recurrences were 4.2%. There was no LR in the NAC skin. They conclude that oncological outcomes remain similar to previously published SSM series. On the other hand, longer follow-up is sine qua non for more robust findings, as we know from mastectomy series that most of the recurrences occur within 5 years.

In our series, none of the patients with NSM diagnosed with invasive cancer have shown nipple involvement in either intraoperative or final pathological assessment. Interestingly, the majority of patients had a tumour distance to NAC <20 mm. At a median follow-up of 48 months, there was no LR, and only one systemic recurrence was detected. Even though our number of cases is small, our follow-up period is 4 years for NSM group and as we know from mastectomy series that timeframe for the peek hazard of LRR is 2- to 3-years after surgery (30). Thus, we can conclude that 2 cm distance is not a precise criterion to determine the safety distance between tumour and NAC. Larger series and long-term follow-up however are needed to clarify this issue.

Considering the survival analyses, long-term complications and implant or TE loss rates, IBR can be safely applied in patients undergoing SSM or NSM. Furthermore, our results demonstrate that breast cancer patients even with close margins (<20 mm) to NAC who underwent NSM with IBR show excellent disease-free and overall survival. Patients with luminal-A type breast cancer undergoing SSM or NSM with IBR also showed excellent survival outcomes when treated by contemporary multidisciplinary oncological management.

## Figures and Tables

**Table 1:**
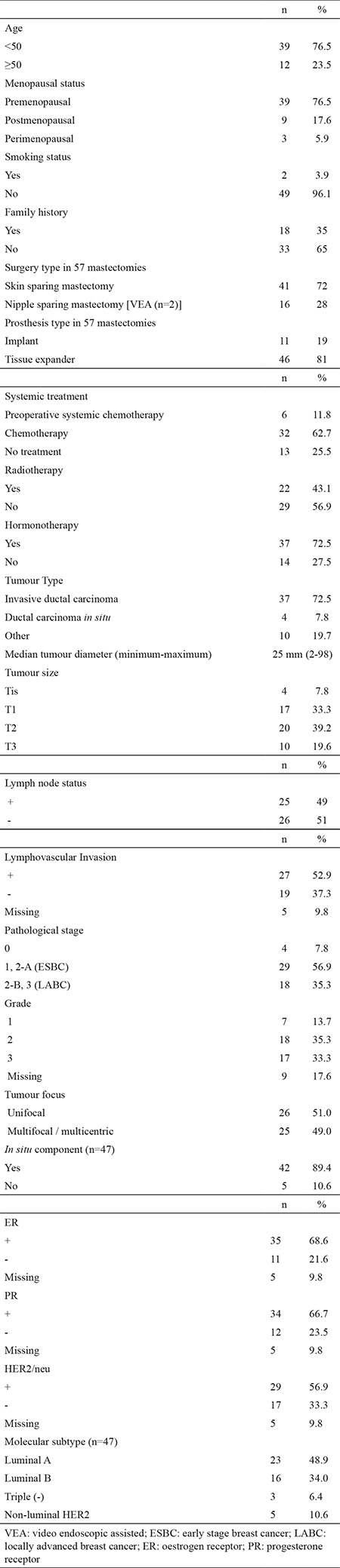
Patient and tumour characteristics, surgical procedure and clinicopatholological features of patients

**Table 2:**
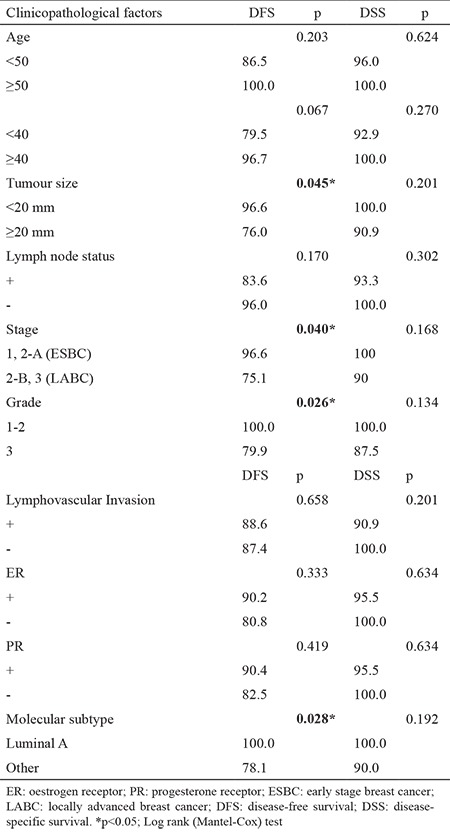
Clinicopathological factors associated with 5-year disease free survival and disease specific survival rates

**Table 3:**
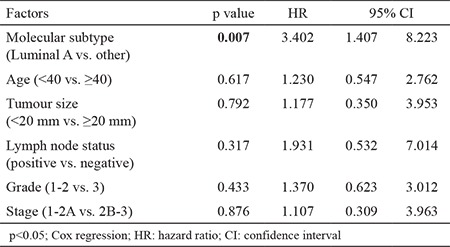
Multivariate Cox regression analysis of prognostic factors

**Table 4:**
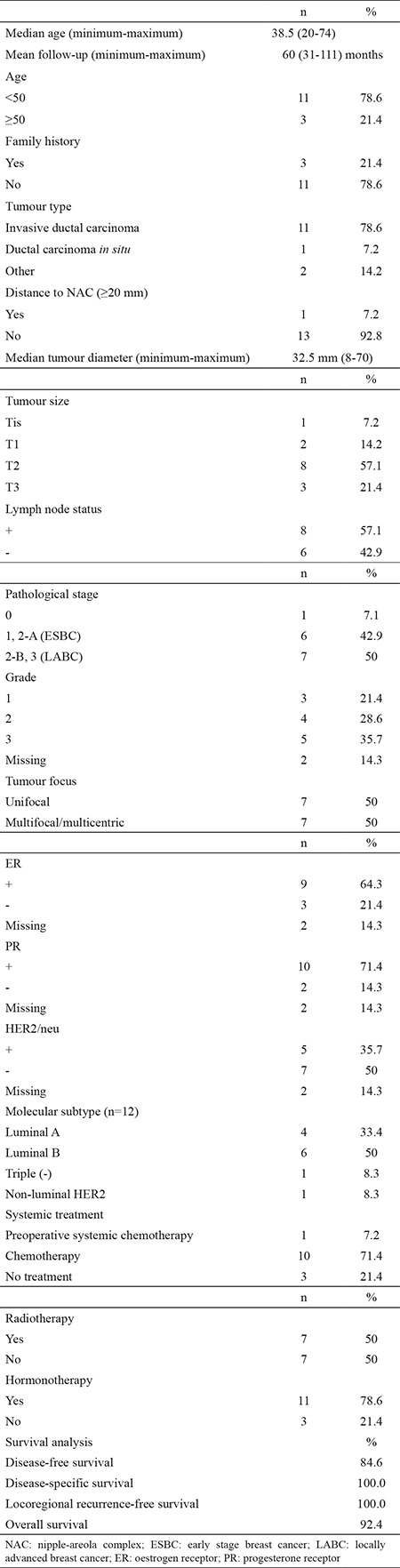
Clinicopathological characteristics and management of patients with nipple-sparing mastectomy (n=14) with survival analysis
